# 3D Nanoarchitecture of Polyaniline-MoS_2_ Hybrid Material for Hg(II) Adsorption Properties

**DOI:** 10.3390/polym12112731

**Published:** 2020-11-17

**Authors:** Hilal Ahmad, Ibtisam I. BinSharfan, Rais Ahmad Khan, Ali Alsalme

**Affiliations:** 1 Division of Computational Physics, Institute for Computational Science, Ton Duc Thang University, Ho Chi Minh City 700000, Vietnam; hilalahmad@tdtu.edu.vn; 2 Faculty of Applied Sciences, Ton Duc Thang University, Ho Chi Minh City 700000, Vietnam; 3 Department of Chemistry, College of Science, King Saud University, Riyadh 11451, Saudi Arabia; ibtisam.i.sh@hotmail.com (I.I.B.); krais@ksu.edu.sa (R.A.K.)

**Keywords:** toxicity, polyaniline, mercury, adsorption, MoS_2_

## Abstract

We report the facile hydrothermal synthesis of polyaniline (PANI)-modified molybdenum disulfide (MoS_2_) nanosheets to fabricate a novel organic–inorganic hybrid material. The prepared 3D nanomaterial was characterized by field emission scanning electron microscopy, high-resolution transmission electron microscopy, energy-dispersive X-ray spectroscopy and X-ray diffraction studies. The results indicate the successful synthesis of PANI–MoS_2_ hybrid material. The PANI–MoS_2_ was used to study the extraction and preconcentration of trace mercury ions. The experimental conditions were optimized systematically, and the data shows a good Hg(II) adsorption capacity of 240.0 mg g^−1^ of material. The adsorption of Hg(II) on PANI–MoS_2_ hybrid material may be attributed to the selective complexation between the–S ion of PANI–MoS_2_ with Hg(II). The proposed method shows a high preconcentration limit of 0.31 µg L^−1^ with a preconcentration factor of 640. The lowest trace Hg(II) concentration, which was quantitatively analyzed by the proposed method, was 0.03 µg L^−1^. The standard reference material was analyzed to determine the concentration of Hg(II) to validate the proposed methodology. Good agreement between the certified and observed values indicates the applicability of the developed method for Hg(II) analysis in real samples. The study suggests that the PANI–MoS_2_ hybrid material can be used for trace Hg(II) analyses for environmental water monitoring.

## 1. Introduction

Mercury (Hg(II)) is one of the most toxic metal pollutants found in the environment and ranks third after arsenic and lead in the National Priorities List of the Agency for Toxic Substances and Disease Registry (ATSDR) [1,2,3]. The Hg(II) contamination of ground and surface water results from geochemical reactions and anthropogenic activities such as improper dumping of electronic waste, thermometer, barometer and mercury lamp waste. Human exposure to metal ions, including Hg(II), can occur during occupational activities, mainly through inhalation and dermal routes in mining and industry, and over a lifetime, from water and food consumption and exposure to soil, dust and air [4,5]. Long-term consumption of drinking water contaminated with Hg(II) can be associated with increased risk of cancers, reproductive problems, detrimental effects on the human brain, blood circulation, immune and reproductive systems and cardiovascular disease [2,6,7]. Therefore, to minimize these risks, the United States Environmental Protection Agency (USEPA) has set the maximum permissible limit of 2 µg L^−1^ [8].

Modern analytical techniques such as X-ray fluorescence, atomic absorption spectrometry, inductively coupled plasma atomic emission spectrometry, and inductively coupled plasma mass spectrometry have been widely used for the analysis of Hg(II) [9,10,11]; however, direct determination of Hg(II) in real aqueous samples is challenging due to their low concentrations and complexity of sample matrices [12]. Therefore, preliminary extraction and preconcentration steps are often necessary before instrumental determination. Various separation methods such as solvent extraction, hydride generation, electro-coagulation, precipitation, cloud point extraction and solid-phase extraction (SPE) are employed to extract metal ions [13,14,15,16,17]. SPE is a preferred procedure because of its advantages such as easy operation, the negligible use of organic solvents, complete desorption of analytes, high preconcentration factor, and used in both batch and column modes [18,19]. Adsorption of the analyte onto nanomaterials in SPE is considered an efficient process based on factors like the high surface area of sorbent, efficient adsorption capacity, and easy functionalize activity [20,21,22,23]. Nanomaterial-based adsorbents have been extensively researched in the past two decades to find new solutions or to enhance the existing solutions in environmental water remediation [21,24,25,26]. In recent years, two-dimensional (2D) nanostructures such as metal chalcogenides, metal hydroxides, and double-layered metal hydroxides have attracted tremendous interest due to their high surface area and a porous structure with large surface active sites [27,28,29,30,31,32]. However, the critical drawback of directly employing these 2D materials in the SPE column is its small size and dispersion in aqueous media, leading to loss of adsorbent during a column operation. Moreover, for the effective deployment of 2D nanostructures, they must prevent stacking. The weak interlayer bonding and low free spacing cause the stacking of nanosheets in the SPE column.

In the present work, we fabricate a blend of 3D hybrid material (organic–inorganic composite) made from 2D MoS_2_ and a 1D polymer polyaniline (PANI) via in situ oxidative polymerization of PANI with exfoliated MoS_2_ nanosheets to overcome the limitations mentioned above. The integration of MoS_2_ nanosheets with PANI restricts the nanosheets leaching from the column and provide stability in aqueous media. Wang et al. reported the polyaniline/zirconium composite to remove organic pollutants [33]. Similarly, Gao et al. reported the hybrid polyaniline/titanium phosphate composite to remove Re(VII) [34]. Moreover, there are no reports on Hg (II) extraction using PANI–MoS_2_ hybrid material. The extensive and profound studies are carried out using PANI–MoS_2_ hybrid nanomaterial to develop a column SPE method for the extraction of trace Hg(II). The accuracy and applicability of the developed method were validated by analyzing the certified reference material and by spiking of real environmental water samples.

## 2. Experimental Details

### 2.1. Materials and Methods

#### 2.1.1. Chemicals and Reagents

All the chemicals used were of analytical grade. Sodium molybdate (Na_2_MoO_4_·2H_2_O), ammonium persulfate ((NH_4_)2S_2_O_8_) and hydrochloric acid were purchased from Thermo Fisher Scientific, New Delhi, India. Thioacetamide (C_2_H_5_NS), silicotungstic acid AR [H(Si(W_3_O_10_)_4_)·xH_2_O] and aniline (C_6_H_5_NH_2_), with 99% purity, were purchased from Sigma Aldrich (Steinem, Germany). A stock solution of divalent mercury ions (Hg(NO_3_)_2_) of 1000 mg L^−1^ was bought from Agilent (Melbourne, Australia) and used after successive dilutions. A 1 M of HNO_3_ and NaOH solution was used to adjust the sample pH.

#### 2.1.2. Synthesis of PANI–MoS_2_ Hybrid Material

The PANI–MoS_2_ hybrid material was synthesized in two steps. In the first step, MoS_2_ nanosheets were hydrothermally synthesized. Briefly, 0.2 mM of sodium molybdate, 1.8 mM of thioacetamide and 5.6 mM of silicotungstic acid were dissolved in 100 mL of deionized water. The reaction mixture was kept in 250 mL of Teflon-coated hydrothermal assembly and heated at 220 °C for 24 h using an air oven. The obtained MoS_2_ nanosheets (0.2 g) were ultrasonicated using probe sonicator in 20 mL of deionized water for 40 min at 27 °C. In the second step, the in situ oxidative polymerization of aniline monomers was carried out onto presynthesized MoS_2_ nanosheets using ammonium persulfate oxidizer. In this process, 4 mL of aniline monomer, 6 mL of HCl and 40 mL of deionized water were stirred together and refrigerated for three h. The cooled reaction solution was added to the exfoliated (ultrasonicated) MoS_2_ nanosheets solution. The formed suspension was stirred in an ice bath (−5 °C) for 30 min. Finally, 10 mL of ammonium persulfate (0.2 M) was added dropwise in the suspension and continuously stirred for 3 h. The obtained solution was filtered, and the residue was washed with deionized water and ethanol. The residue was dried in a vacuum at 60 °C for 12 h. The obtained PANI–MoS_2_ hybrid material was characterized and studied for Hg(II) adsorption properties.

### 2.2. Material Characterization

The surface morphology and structural properties were observed using a scanning electron microscope (FE-SEM, Zeiss, Sigma, Tokyo, Japan) and high-resolution transmission electron microscopy (HR-TEM F30 S-Twin TECNAI FEI, Tokyo, Japan) operating at an acceleration voltage of 300 kV. Samples for HRTEM characterization were prepared by dispersing the material powder into ethanol by ultrasonic treatment. Rigaku Smart Lab X-ray diffractometer with Cu K_α_ radiation at 1.540 Å in the 2θ range of 20–90° is used to study crystal structure and phase determination. The Brunauer–Emmett–Teller (BET) surface area measurements were carried out using an Autosorb-iQ one-station (Quantachrome Instruments, Boynton Beach, FL, USA). The nitrogen gas was used for sorption and desorption analysis at low relative pressures. The surface charge of the materials was investigated by Zeta potential (z) measurements on a Zetasizer (Malvern Instruments, Malvern, UK). A Shimadzu TGA-50 thermal analyzer was used to conduct thermal gravimetric analysis (TGA) at a heating rate of 10 °C/min from 27 °C to 650 °C. A Perkin Elmer inductively coupled plasma optical emission spectrometer (ICP-OES model Avio 200, Melbourne, Australia) was used to analyze the Hg(II) concentrations. The ATR-IR (attenuated total reflectance infrared spectroscopy) (Vertex 70v, Bruker, Ettlingen, Germany) analysis of PANI–MoS_2_ adsorbent, before and after Hg(II) adsorption, were carried out in the range of 400–4000 cm^−1^ (with the accumulation of 60 scans). The surface elemental analysis was carried out using X-ray photoelectron spectroscopy (XPS, Thermo Fisher Scientific ESCALAB 250Xi, Waltham, MA, USA). The studies were performed in a binding energy range of 0–1400 eV. MgK alpha was used as an X-ray source at 1253.6 eV with a detection angle of 45° and a depth of 10 nm.

### 2.3. Recommended Column Procedure

A polytetrafluoroethylene column (Length = 10 cm; diameter = 1 cm) (Merck, Shanghai, China) packed with 0.5 g of PANI–MoS_2_ hybrid material (bed height = 1.6 cm) was used for the column through experiments. A bench of model solutions (100 mL) of desired Hg(II) concentration maintained at pH 6.0 using 1 M of HNO_3_ and NaOH solution were percolated through the column bed at a flow rate of 8 mL min^−1^ using a peristaltic pump (Scenchen, Hebei, China). The adsorbed Hg(II) was stripped out using a 5 mL of 0.5 M HCl, and the concentration of adsorbed Hg(II) was analyzed by ICP-OES.

## 3. Results and Discussion

### 3.1. Characterization

**Figure 1:**
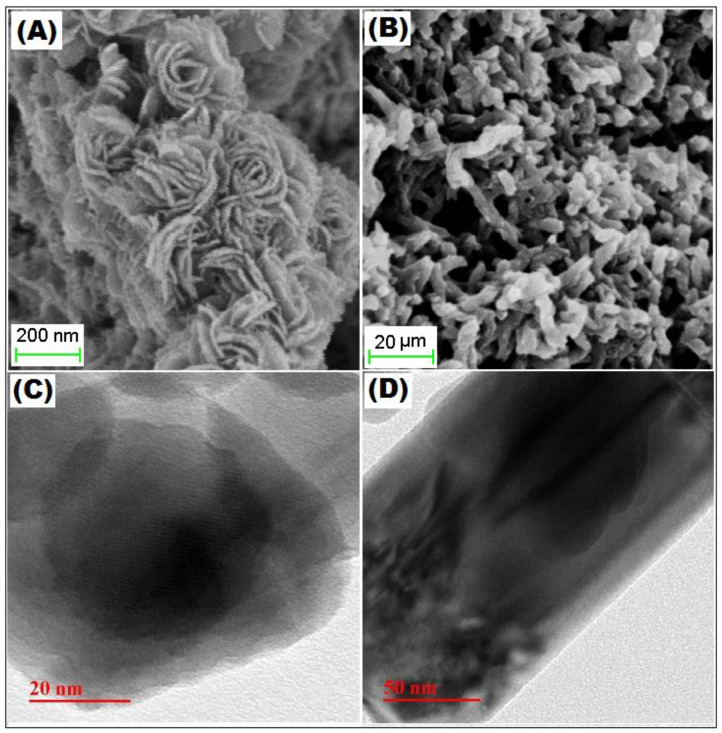
Scanning electron microscope image of (**A**) bare MoS_2_; (**B**) polyaniline/molybdenum disulfide hybrid nanomaterial (PANI-MOS_2_); and transmission electron microscopy image of (**C**) MoS_2_; and (**D**) PANI-MOS_2_.

**Figure 2:**
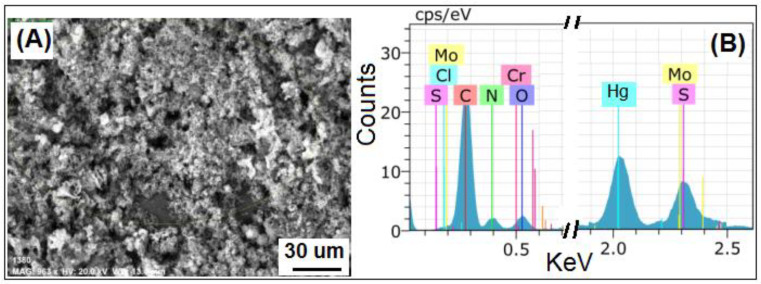
PANI–MoS_2_ after Hg(II) adsorption (**A**) FESEM image (**B**) EDX spectra.

**Figure 3:**
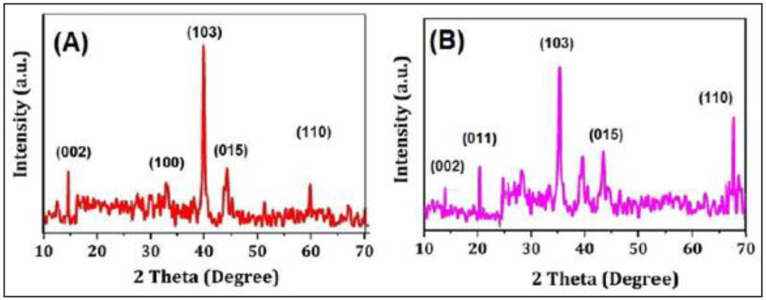
XRD diffraction pattern of (**A**) MoS_2_; and (**B**) PANI-MoS_2_.

**Figure 4:**
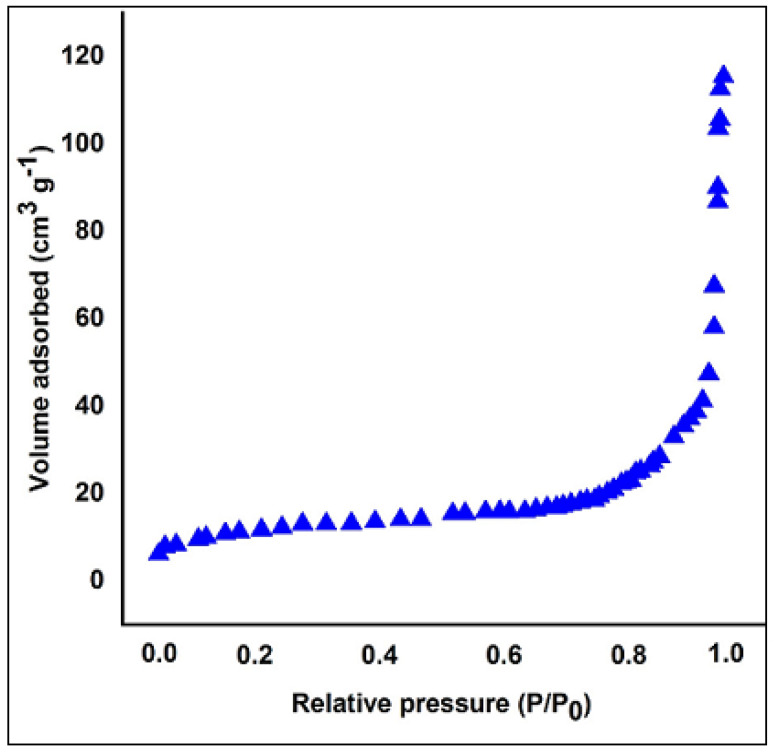
Nitrogen adsorption isotherm of PANI–MoS_2_ adsorbent.

**Figure 5:**
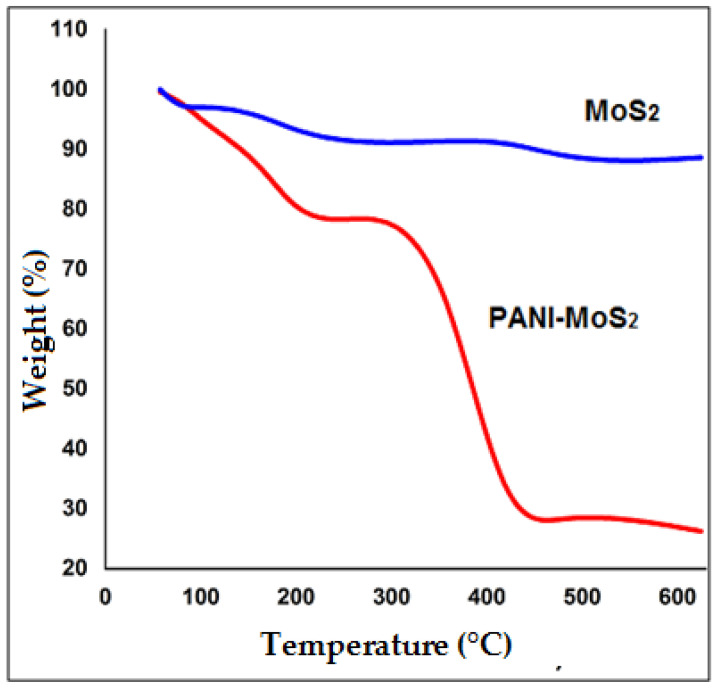
TGA profile of bare MoS_2_ and PANI–MoS_2_.

**Figure 6:**
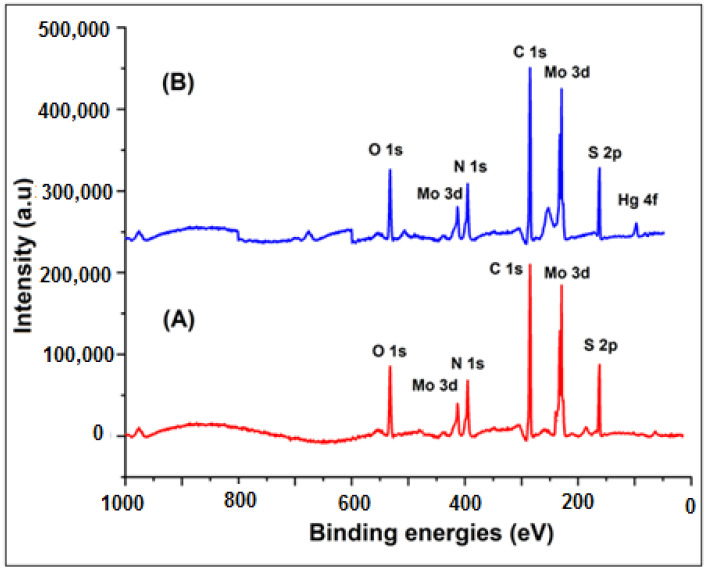
X-ray photoelectron spectra of PANI–MoS_2_ (**A**) before Hg(II) adsorption and (**B**) after Hg(II) adsorption.

The surface morphology of MoS_2_ and PANI–MoS_2_ hybrid composite is shown in Figure 1A,B. Figure 1A shows the MoS_2_ nanosheets arranged in a flower-like structure with porous morphology. Figure 1B shows that PANI uniformly bounded the MoS_2_ sheets. The resulting PANI–MoS_2_ structure had a long tube-like morphology with a rough surface due to constituted nanoparticles, indicating that the PANI–MoS_2_ may provide additional binding sites for Hg(II) adsorption. The difference in the HRTEM images of Figure 1C,D reveals that the PANI was successfully immobilized on MoS_2_ nanosheets. From Figure 1C,D, the two contrasted regions, the dark region representing MoS_2_ nanosheets, nearby many ultrathin single MoS_2_ nanosheets, were also present, and the lighter region represents PANI nanofibers. Figure 2A,B illustrates the SEM and EDX spectra of PANI–MoS_2_ after Hg(II) adsorption. Figure 3A,B shows the X-ray diffraction (XRD) pattern of MoS_2_ and PANI–MoS_2_. The diffraction peaks observed at 2θ = 13.10, 32.70, 35.15, 41.50 and 59.50 corresponds to the (002), (100), (103), (015) and (110) planes of MoS_2_ (Figure 3A). The d-spacing of MoS_2_ calculated using Bragg’s law was found to be 6.71 Å. From the XRD data (Figure 3B), the interlayer spacing of MoS_2_ nanosheets in PANI–MoS_2_ hybrid material was found to be 6.24 Å. The observed data depicted that the aniline forms mono and bilayers structures on MoS_2_ and the polymerization of intercalated aniline monomer reduces the interlayer distance from 6.71 to 6.24 Å. It was suggested that the polymerization of aniline occurs outside the MoS_2_ nanosheets. Also, the PANI–MoS_2_ hybrid material was less crystalline than bare MoS_2_ attributes to flexible PANI–MoS_2_ hybrid structure with an amorphous surface. The nitrogen gas adsorption–desorption analysis was carried out to characterize the physical properties of the adsorbent; the nitrogen isotherms are shown in Figure 4. The average surface area calculated by the Brunauer–Emmett–Teller (BET) method was found to be 29.0 m^2^ g^−1^. The thermal analysis of PANI–MoS_2_ under air atmosphere was carried out to study the thermal stability. It was observed that the material has thermal stability, up to a temperature of 320 °C (Figure 5). The TGA shows minor weight loss around 100–120 °C, which may occur due to interlayer water content loss. The major weight loss commences at 320–600 °C may be attributed to the oxidative degradation of the polyaniline component of the PANI–MoS_2_ hybrid material. The ATR-IR spectra of PANI–MoS_2_ before and after Hg(II) adsorption is shown in Supplementary Materials Figure S1. The peaks observed at 1600, 1485, 1290 and 1150 cm^−1^ in the spectra of PANI–MoS_2_ were attributed to the stretching vibrations of the C–C ring, C–H bending and C–N stretching vibrations of the quinoid and benzenoid ring of PANI, respectively. The characteristic MoS_2_ peak was observed at 468 cm^−1^. The small peak observed at 798 cm^−1^ was corresponds to S-S stretching vibration. After Hg(II) adsorption, the weak intensity peak observed at 450 cm^−1^ may be attributed to Hg–S stretching vibration. The elemental composition of PANI–MoS_2_ was further examined by XPS analysis. Figure 6A,B shows the XPS survey of PANI–MoS_2_ before and after Hg(II) adsorption. In Figure 6A, the peaks at binding energies of 162.0, 229.1, 285.0, 395.0 and 532 eV correspond to S 2p, Mo 3d, C 1s, N 1s and O 1s, respectively. In Figure 6B, the presence of Hg 4f peak at a binding energy of 100.6 eV attributes to the adsorption of Hg(II) onto PANI–MoS_2_ adsorbent.

### 3.2. Optimized Sample pH and Adsorption Mechanism

**Figure 7:**
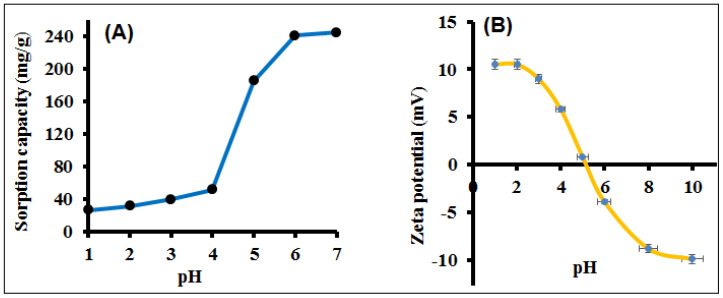
(**A**) Effect of sample pH on the adsorption of Hg(II); (**B**) zeta potential of PANI-MOS_2_ adsorbent (experimental conditions: sorbent amount 0.5 g; sample volume 100 mL; flow rate 8 mL min^−1^, Hg^2+^ 100 mg L^−1^).

The solution pH plays an essential role in the adsorption of the analyte by influencing the surface charge of adsorbent and metal ion species distribution. Optimum pH can reduce the interferences caused by the sample matrix and improves the method selectivity. Therefore, the optimization of sample pH is the first step. The adsorption of Hg(II) on PANI–MoS_2_ was studied in the pH range of 1.0–7.0. Basic sample pH (pH 8.0 to 10.0) was avoided due to the formation of Hg precipitates. A bench of model solutions (volume 100 mL), each containing 100 ppm of Hg(II) maintained at pH 1.0–7.0 (using 1 M of HNO_3_ and NaOH solution), was passed through columns packed with 0.5 g of PANI–MoS_2_ hybrid material. The adsorbed Hg(II) was eluted and subsequently determined by ICP-OES. As shown in Figure 7A, the PANI–MoS_2_ hybrid material shows Hg(II) adsorption at a wider pH range. It can be seen that the Hg(II) adsorption at low pH values (up to pH 3) was not much affected and increased quickly after pH 4 and reached a maximum at pH 6.0–7.0. A complete recovery ca. 100% was observed at pH 6.0–7.0. The adsorption of Hg(II) mainly occurs on the active sites of PANI–MoS_2_ composite via favorable chelation of Hg(II) with sulfide ions of PANI–MoS_2_, in addition to the amine and imine functionalities of PANI. The intrinsic sulfur ions of PANI–MoS_2_ hybrid material are the primary binding sites for the adsorption of Hg(II). At low pH values, the PANI–MoS_2_ hybrid material shows less adsorption of Hg(II) due to the protonation of active/binding sites. At higher sample pH, the–S ions get deprotonated, and the soft-soft interaction between the -S ions and Hg(II) dominates thereby, increases the Hg(II) adsorption [35,36]. To better understand such observations, the surface charge of PANI–MoS_2_ was measured (Figure 7B). For comparison, the zeta potential of nascent MoS_2_ and PANI were also presented in Figure S2. The results of zeta potential indicate that at pH values 1.0–5.0, the PANI–MoS_2_ surface was positively charged, resulting in weaker interaction between the surface groups and Hg(II) and above pH 5.0, the presence of negative charge on the surface of PANI–MoS_2_ hybrid material, leading to the efficient adsorption of Hg(II) which is appropriate following the adsorption results (Figure 7A). In conclusion, the chelation of Hg(II) with the -S ions of PANI–MoS_2_ hybrid material and the electrostatic interactions are the primary adsorption mechanisms for Hg(II); thus, pH 6.0 was chosen for the adsorption of Hg(II) in further experiments.

### 3.3. Preconcentration and Breakthrough Studies

**Figure 8:**
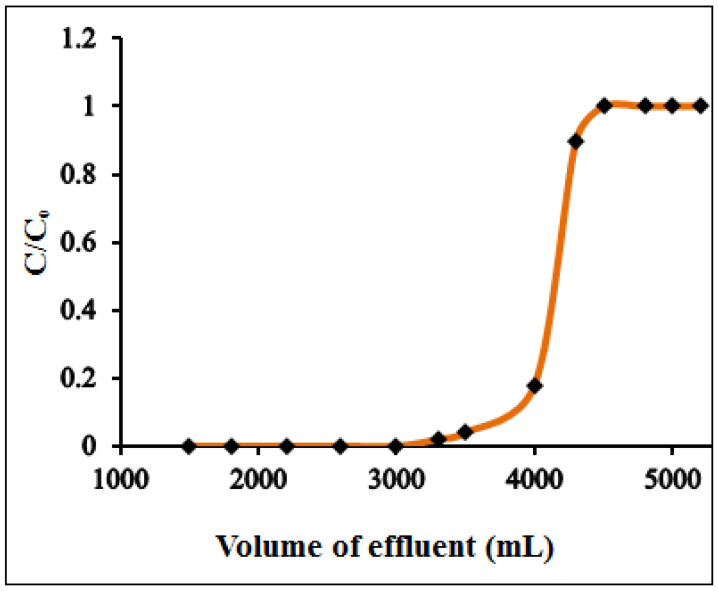
Breakthrough curve for the adsorption of Hg(II) (experimental conditions: sorbent amount 0.5 g; pH = 6.0; flow rate = 8 mL min^−1^, Hg^2+^ 10 mg L^−1^).

Due to the ultra-low concentration of Hg(II) ions, direct instrumental determination of Hg(II) contamination level in surface and ground waters is challenging. Therefore, a preconcentration technique is a prerequisite to improve the analyte concentration by transforming it from a large sample volume to a smaller one. To analyze the preconcentration limit and preconcentration factor of the developed method, a series of model solutions with varying sample volume (1500–4000 mL), each contains a fixed amount of 1.0 µg of Hg(II) and maintained at pH 6.0, were passed through the column at a flow rate of 8 mL min^−1^. The sorbed Hg(II) was then eluted using a suitable eluting agent, and the amount of Hg(II) was determined by ICP-OES. Table 1 illustrated the obtained results. It was observed that the quantitative recovery of Hg(II) was achieved within a sample volume of 3200 mL while on increasing the sample volume to 3500–4000 mL, the percent recovery of Hg(II) noticeably decreased to 90–85%. Thereby, a high preconcentration limit of 0.31 µg L^−1^ was obtained with a preconcentration factor of 640. Such a high preconcentration factor is necessitated for column preconcentration of trace metal ions. A 5000 mL of sample volume containing 10 mg L^−1^ of Hg(II) was passed through the column under optimum conditions to study the breakthrough curve. The fractions of effluent were collected at certain time intervals and analyzed by ICP-OES. Figure 8 shows the breakthrough curves for the analyte ion. The breakthrough volumes for Hg(II) at which the analyte concentration is about 3–5% of initial metal concentration were found to be 4000 mL. The breakthrough capacity obtained is very close to the column adsorption capacity, suggesting the potential application of PANI–MoS_2_ adsorbent for continuous column operation.

### 3.4. Amount of Sorbent and Choice of Eluent and Concentration

**Figure 9:**
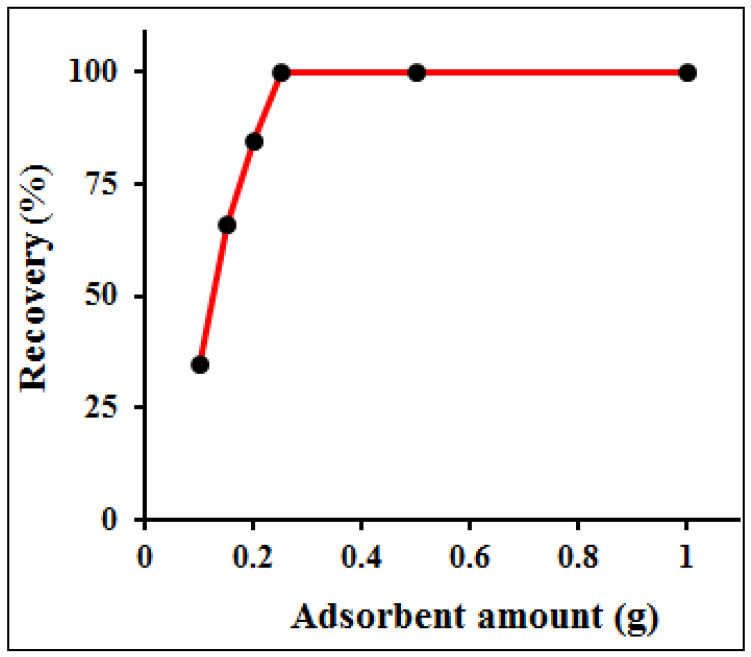
Effect of adsorbent amount on the adsorption of Hg(II) (experimental conditions: sample volume 100 mL; pH = 6.0; flow rate 8 mL min^−1^; Hg^2+^ 10 mg L^−1^).

**Figure 10:**
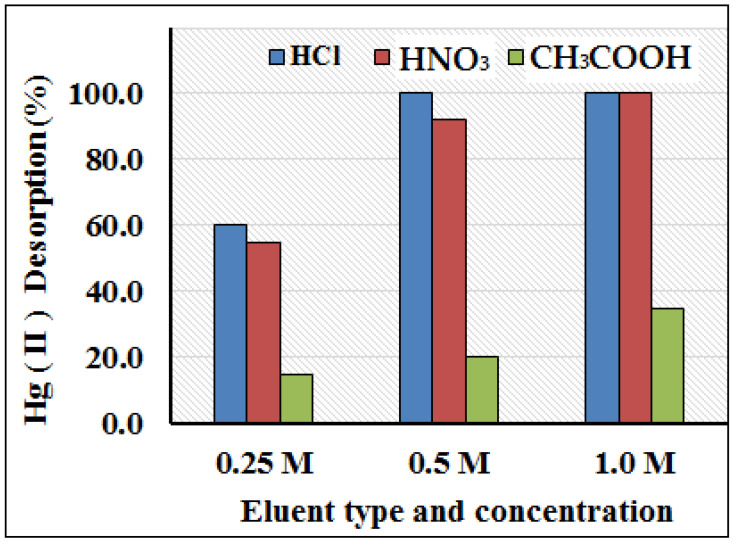
Effect of type and concentration of eluting agents on the desorption of Hg(II) (experimental conditions: sample volume 100 mL; pH = 6.0; flow rate 8 mL min^−1^; Hg^2+^ 100 mg L^−1^).

The effect of adsorbent dosage on the column preconcentration of Hg(II) was investigated from 0.1 to 1.0 g of the PANI–MoS_2_-packed column. A model solution of Hg(II) (sample vol. 100 mL; Hg^2+^ = 10 mg L^−1^) was passed through the column, following the optimized experimental conditions. It was observed that by increasing the adsorbent amount from 0.1 to 0.25 g, the percent recovery of Hg(II) increases and reached 100% at 0.25 g of adsorbent; and remains constant up to 1.0 g of PANI–MoS_2_ (Figure 9). For subsequent experiments, 0.5 g of adsorbent was optimized for the rest of the experiments. The complete desorption of adsorbed metal ions using a suitable eluent is necessary to reuse the column for the next adsorption cycle. A different eluting agent such as acetic acid, hydrochloric and nitric acids with varying concentration (0.25–1.0 M) and volumes (2–5 mL) was passed through the column with a flow rate of 2 mL min^−1^. The eluent solution of hydrochloric and nitric acids resulted in the varying recovery of Hg(II) (Figure 10); among them, 5 mL of 0.5 M hydrochloric acid at a flow rate of 2 mL min^−1^ suitably desorbed the Hg(II) (recovery > 99.9%) and prepared the column for next adsorption experiments. Therefore, 5 mL of 0.5 M hydrochloric acid at a flow rate of 2 mL min^−1^ was used as eluent for further experiments.

### 3.5. Influence of Column Flow Rate on Preconcentration Efficiency

**Figure 11:**
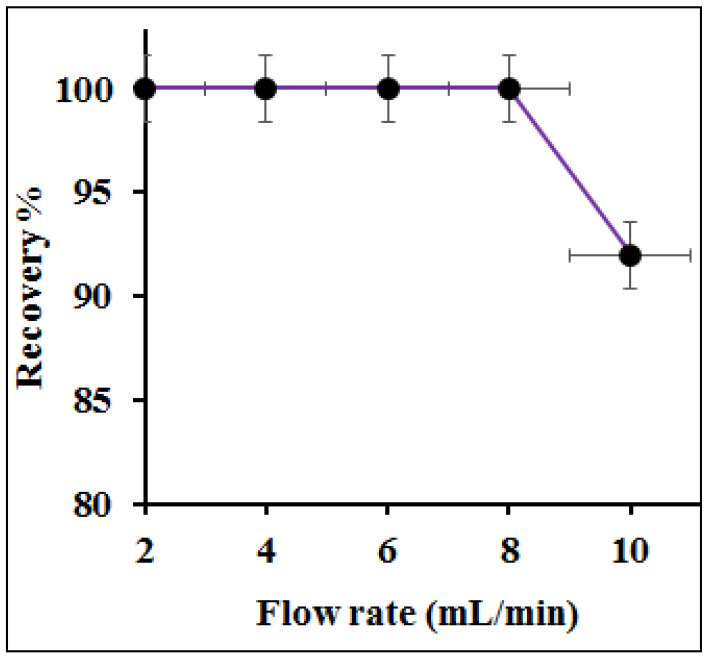
Effect of sample flow on the adsorption of Hg(II) (experimental conditions: sample vol. 100 mL; pH = 6.0; Hg^2+^ 10 mg L^−1^).

The sample flow in the analyte adsorption alters the analyte extraction efficiency and rules the analysis time. Generally, an optimized sample flow permits an equilibrium between the metal ions and the column adsorbent to facilitate the adsorption performance. The effect of flow rate on the adsorption of Hg(II) was investigated by varying sample flow rates from 2 to 10 mL min^−1^ with 100 mL of 10 µg L^−1^ sample solutions at pH 6.0. As shown in Figure 11, the complete recovery of Hg(II) was attained up to a flow of 8 mL min^−1^. On increasing the sample flow to 9 mL min^−1^, 92% of Hg(II) recovery was observed due to insufficient contact between the analyte and active sites of PANI–MoS_2_. Hence, 8 mL min^−1^ of the column flow rate was optimized for the rest of the experiments.

### 3.6. Interference Studies

The effect of co-existing ions such as ferric, nitrate, carbonate, chloride, sulfate, phosphate and heavy metal ions, including alkali and alkaline earth metal in sorption of Hg(II), were investigated, and the observed data were reported in Table 2. The tolerance level of co-ions was studied by passing a model solution (vol. 100 mL; Hg^2+^ conc. 10 µg L^−1^) contained a varying concentration of interfering ions through the PANI–MoS_2_ packed column. The tolerance limit was set as the concentration of co-ions results in a deviation of ±5% in the signal intensity of recovered Hg(II). Under optimum conditions, the proposed method demonstrates fairly good tolerance against co-ions with good recovery of Hg(II) was achieved in the range of 98–100% for quantitative determination.

### 3.7. Analytical Figures of Merit and Method Validation

Analytical method validation has been accounted for irrespective of the applicability of the developed procedure for gaining useful data. Following the optimum experimental parameters, the calibration plot for Hg(II) analysis was obtained in the range of 0.2 to 100 µg L^−1^ of Hg(II), with a good correlation coefficient, R^2^ = 0.9998. The limit of detection (LOD) and limit of quantification (LOQ), obtained as the concentrations equivalent to three times and ten times of the standard deviation of eleven blank runs, were found as 0.06 µg L^−1^ and 0.2 µg L^−1^, respectively [37]. Thus, it allows for the ultra-trace determination of Hg(II) in water samples. The relative standard deviation (RSD) that characterizes the method’s precision, evaluated for eleven replicate samples containing 5 µg L−1 of Hg(II), was found in the range of 3.0–4.5%. The validity of the proposed method was observed by analyzing the standard reference material (SRM 1641d). The results are shown in Table 3. The closeness of measured value with the certified values is in good agreement, indicates the accuracy of the developed method. In addition, the spiking analysis with two levels of Hg(II) concentration was carried out using different environmental water samples such as household water (tap), industrial wastewater and river water samples (Table S1). The recoveries of the added amount of Hg(II) were satisfactorily recovered with a 95% confidence limit, and the mean percentage recoveries range between 99.0% to 100.2%, with an RSD value in the range 0.35–2.26%. This suggests the accuracy of the method to preconcentrate the trace analytes in real water samples for accurate determination.

## 4. Conclusions

A novel organic–inorganic hybrid adsorbent was synthesized by surface modification of bare MoS_2_ using PANI. The prepared PANI–MoS_2_ hybrid material shows selective extraction of Hg(II) in presences of co-existing ions. The fast and selective Hg(II) adsorption may be attributed to the soft acid-soft base interaction between the Hg(II) and–S ions of the PANI–MoS_2_ adsorbent. A comparative data on the Hg(II) adsorption capacity of prepared material with previous literature was compared and is shown in Table 4. The PANI–MoS_2_ adsorbent shows comparable adsorption capacity over the previously reported nanoadsorbents. The proposed method’s accuracy was validated by analyzing reference material and the standard addition method (RSD < 5%). The proposed methodology is simple and successfully used in the quantitative analyses of trace Hg(II) to monitor the Hg(II) level in real environmental water samples.

## Figures and Tables

**Table 1:** Analytical data of preconcentration and breakthrough studies (column parameters: sample pH 6; flow rate 8 mL min^−1^; eluent vol. 5 mL; sorbent amount 0.25 g).

		Preconcentration Studies				Breakthrough Studies	
Sample Volume (mL)	Hg(II) Amount (µg L^−1^)	E(%) ^a^	PL ^b^ (µg L^−1^)	PF ^c^	Column Adsorption Capacity (mg g^−1^)	Breakthrough Volume (mL)	Breakthrough Capacity (mg g^−1^)
1500	0.66	100	0.66	300		4000	160.5
2000	0.50	100	0.50	400	240.0		
2700	0.37	100	0.37	540			
3200	0.31	100	0.31	640			
3500	0.29	90	-	-			
4000	0.25	85	-	-			

^a^ Extraction percentage; ^b^ Preconcentration Limit; ^c^ Preconcentration Factor.

**Table 2:** Interference studies on the adsorption of analyte ions (experimental conditions: M^n+^ = 100 µg L^−1^, sample volume = 100 mL, pH = 6.0, flow rate 8 mL min^−1^, eluent 5 mL of HCl; N = 3).

Interfering Ions	Salt Added	Amount Added (×10^3^ µg L^−1^)	Recovery % (RSD)
			Hg(II)
Na^+^	NaCl	6000	98.0 (4.15)
K^+^	KCl	5600	98.9 (4.65)
Ca^2+^	CaCl_2_	900	97.0 (3.00)
Mg^2+^	MgCl_2_	1500	99.7 (4.00)
Cl^−^	NaCl	9000	100 (4.23)
Br^−^	NaBr	8000	99.8 (3.54)
CO_3_^2−^	Na_2_CO_3_	4500	98.7 (4.18)
SO_4_^2−^	Na_2_SO_4_	4200	98.6 (4.25)
NO_3_^−^	NaNO_3_	3500	100.4 (4.05)
CH_3_COO^−^	CH_3_COONa	4000	96.5 (4.94)
C_6_H_5_O_7_^3−^	Na_3_C_6_H_5_O_7_	3300	99.5 (4.16)

**Table 3:** Analytical method validation by analyzing standard reference material (SRM) after column preconcentration (column conditions: sample volume 100 mL, flow rate 8 mL min^−1^, eluent 5 mL HCl, sorbent amount 0.5 g).

Samples	Analyte	Certified Values (µg g^−1^)	Values Found by Proposed Method (µg g^−1^) ^a^ ± Standard Deviation	Value of *t*-Test ^b^
NIST SRM 1641d	Hg(II)	1.56 ± 0.02	1.55 ± 0.06	1.37

^a^ Mean value, N = 3; ^b^ at 95% confidence level.

**Table 4:** Hg(II) adsorption capacities of different nanomaterials based on previous literature.

Adsorbent	Metal Ion	Adsorption Capacity (mg g^−1^)	References
PANI–MoS_2_	Hg(II)	240	This work
MOF	Hg(II)	627.6	[38]
Fe_3_O_4_@SiO_2_SH	Hg(II)	132.0	[39]
MSCFM	Hg(II)	160.4	[12]
Titanate nanoflowers	Hg(II)	454.5	[40]
Magnetic composite	Hg(II)	149.3	[41]

## Supplementary Materials

The following are available online at https://www.mdpi.com/2073-4360/12/11/2731/s1, Figure S1: Zeta potential envelope of bare PANI and bare MoS_2_, Figure S2; ATR-IR spectra of PANI-MoS_2_ before and after Hg(II) adsorption, Table S1: Solid phase extraction and preconcentration of trace Hg(II) in real samples analyses after to determine Hg(II) concentration by ICP-OES (column conditions: sample volume 250 mL, flow rate 8 mL min^−1^, eluent 5 mL HCl, sorbent amount 0.25 g).
