# Supplementary material for: 3D Nanoarchitecture of Polyaniline-MoS2 Hybrid Material for Hg(II) Adsorption Properties
Source: Polymers (Basel). 2020 Nov 17;12(11):2731. doi: 10.3390/polym12112731 (PMC7698604; doi:10.3390/polym12112731)
Supplement: Supplementary file 1 [file polymers-12-02731-s001.pdf]

3D nano-architect of polyaniline-MoS_2 hybrid material for Hg(II) adsorption properties

Hilal Ahmad [1,2], Ibtisam I. BinSharfan [3], Rais Ahmad Khan [3] and Ali Alsalme [3,*]

[1] Division of Computational Physics, Institute for Computational Science, Ton Duc Thang University, Ho Chi Minh City 700000, Vietnam; hilalahmad@tdtu.edu.vn

[2] Faculty of Applied Sciences, Ton Duc Thang University, Ho Chi Minh City 700000, Vietnam

[3] Department of Chemistry, College of Science, King Saud University, Riyadh 11451, Saudi Arabia; ibtisam.i.sh@hotmail.com (I.I.B.); krais@ksu.edu.sa (R.A.K.)

* Correspondence: aalsalme@ksu.edu.sa

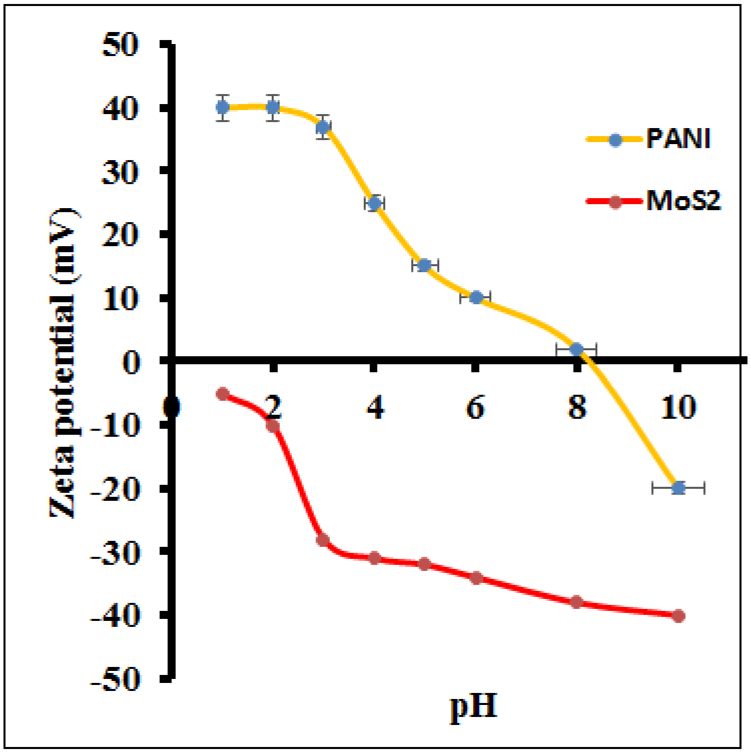

Figure S1: Zeta potential envelope of bare PANI and bare MoS_2.

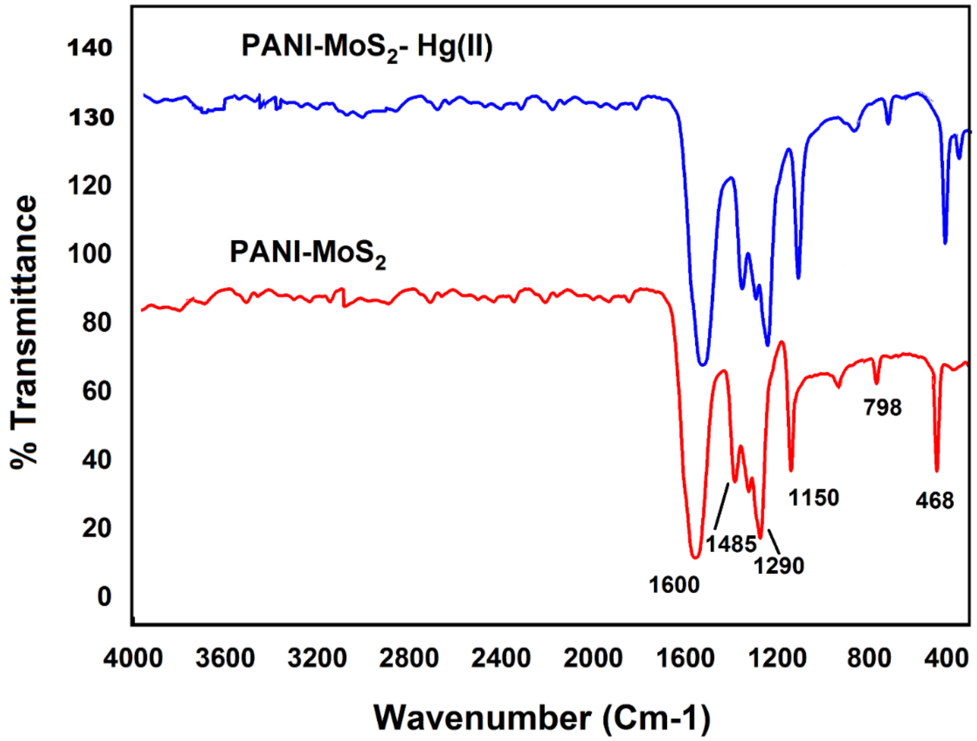

Figure S2: ATR-IR spectra of PANI-MoS_2 before and after Hg(II) adsorption.

Table S1: Solid phase extraction and preconcentration of trace Hg(II) in real samples analyses after to determine Hg(II) concentration by ICP-OES (column conditions: sample volume 250 mL, flow rate 8 mL min^{-1}, eluent 5 mL HCl, sorbent amount 0.25 g).

Samples	Spiked amount (μg)	Hg(II) found (μg L^{-1}) ± standard deviation[a]	Recovery percentage of added amount (RSD)[c]	value of t-test[d]
Tap water	0	ND[b]	-	-
	5	4.98 ± 0.44	99.6 (0.37)	0.76
	10	9.97 ± 0.52	99.7 (0.42)	1.28
River water	0	5.5	-	-
	5	10.48 ± 0.70	99.6 (0.35)	1.56
	10	15.58 ± 0.86	100.8 (2.26)	1.77
Industrial wastewater	0	12.80 ± 1.42	-	1.64
	5	17.75 ± 1.15	99.0 (0.68)	1.87
	10	22.82 ± 1.42	100.2 (0.97)	2.64

[a] N=3; [b] not detected; [c] Relative standard deviation; [d] at 95% confidence level, $t_{critical}$ = 4.303
